# Affinity Based Nano-Magnetic Particles for Purification of Recombinant Proteins in Form of Inclusion Body

**DOI:** 10.29252/ibj.24.3.192

**Published:** 2019-12-07

**Authors:** Masoud Seyedinkhorasani, Reza Ahangari Cohan, Saeid Taghavi Fardood, Farzin Roohvand, Dariush Norouzian, Malihe Keramati

**Affiliations:** 1 Nano-Biotechnology Department, Pasteur Institute of Iran, Tehran, Iran;; 2 Department of Chemistry, University of Zanjan, Zanjan, Iran;; 3 Virology Department, Pasteur Institute of Iran, Tehran, Iran

**Keywords:** Inclusion body, His-tag, Magnetic nanoparticle, Protein purification

## Abstract

**Background:**

Protein purification is the most complicated issue in the downstream processes of recombinant protein production; therefore, improved selective purification methods are important. Affinity-based protein purification method using His-tag and Ni-NTA resins is one of the most common strategies. MNPs can be used as a beneficial alternative for Ni-NTA resins. However, there is no data on the capability of MNPs for protein purification from inclusion bodies; this issue is studied here.

**Methods:**

Recombinant His-tagged proteins of EGFP-His and SK-His were expressed in *E. coli* BL-21 (DE3) in soluble and inclusion body forms, respectively. MNPs including Fe_3_O_4_ magnetic core, SiO_2_ shell, and Ni^2+^ on the surface were synthesized by sol-gel and hydrothermal reactions and then characterized by XRD, VSM, and SEM imaging. Both synthesized Fe_3_O_4_@NiSiO_3_ and Fe_3_O_4_@Ni_x_SiO_y _MNPs were employed to purify EGEP-His and SK-His under native and denaturing conditions, respectively. The quantity and purity of purified proteins were analyzed by micro-Bradford assay and SDS-PAGE, respectively.

**Results:**

Both synthesized MNPs were spherical and well-dispersed with the size ranging from 290 to 415 nm. Synthesized MNPs contained Fe_3_O_4,_ SiO_2_ shell, and Ni^2+^ on their structures with suitable magnetization properties. Using Fe_3_O_4_@NiSiO_3 _and Fe_3_O_4_@Ni_x_SiO_y _yielded 192 and 188 µg/mg of SK-His, as compared to 207 and 195 µg/mg of EGFP-His, respectively.

**Conclusion:**

MNPs containing magnetic Fe_3_O_4 _core, SiO_2_ shell, and Ni^2+^on their surface are versatile alternatives for Ni-NTA resins in protein purification for proteins expressed in both soluble and inclusion body forms.

## INTRODUCTION

Protein purification is the main step of downstream processing of recombinant protein production that might impose a load of more than half of the total process cost^[^^1^^]^. Therefore, development of rapid and efficient methods for purification of target proteins from cell extracts remains as an important issue. Currently, affinity chromatography based on fusion affinity tag, which is co-expressed with the target protein, is one of the well-developed techniques for protein separation and purification. In affinity-based purification method, a variety of fusion affinity tags, such as chitin binding domain, maltose binding protein, FLAG-tag, S-tag, and His-tag and their immobilized ligands, have been developed^[^^2^^,^^3^^]^. In spite of the simplicity of His-tagged protein purification on column chromatography, it bears some limitations, including pretreatment steps to wipe out the cell debris, time-consuming process, and difficult manipulations^[^^2^^,^^4^^]^. Recently, new separation methods have been developed for purification of His-tagged protein based on MNPs^[^^5^^-^^8^^]^. MNPs are biocompatible nanostructures with high surface area to volume ratio and represent rapid and efficient protein separation traits^[^^5^^,^^8^^,^^9^^]^. Several ionic moieties and groups of compounds, including Fe_3_O_4_/IDA-Cu^+2[^^10^^]^, Fe_3_O_4_/SiO_2_-GPTMS-Asp-Co^[^^11^^]^, Fe_3_O_4_/Au–ANTA–Co^2+[^^12^^]^, Fe_3_O_4_@NiSiO_3_^[^^6^^]^, and Fe_3_O_4_@Ni_x_SiO_y_^[^^13^^]^) have been coated on the surface of the MNPs and functionalized them for selective protein separation. Chemical stability, biocompatibility, low cost, and the simple synthesis process for silicate and Ni surface coating of these MNPs have frequently been reported^[^^6^^,^^8^^,^^13^^,^^14^^]^.

Although the efficacy of MNPs with silicate shell and Ni coat have been shown for the purification of His-tagged protein models expressed in soluble forms under native conditions, their efficiency in purification of those models in inclusion bodies, under denaturing conditions, remains undetermined^[^^11^^,^^12^^]^. It should be considered that the expression of recombinant proteins in the form of inclusion bodies will increase productivity and facilitate the purification process^[^^15^^,^^16^^]^. In the present study, two different kinds of MNPs, including Fe_3_O_4_@NiSiO_3_ and Fe_3_O_4_@Ni_x_SiO_y_, were synthesized, and the capability for His-tagged protein purification in both inclusion bodies (under denaturing conditions) and soluble forms (under native conditions) were assessed. The protein models of EGFP in soluble form and SK as an inclusion body form were used. 

## MATERIALS AND METHODS

All chemicals used were of analytical grade. 1-Octadecene, NH_3_·H_2_O (25%-28%), TEOS (98%), NaOH, FeCl_3_·6H_2_O, NiCl_2_·6H_2_O (98%), oleic acid, ammonium chloride, and cetyltrimethyl ammonium bromide were obtained from Sigma-Aldrich, USA. Polyethylene glycol 1000 and HCl were purchased from Merck (Germany).

**Synthesis of Fe**_3_ **O**_4_ **nanoparticles**

First, 2.8 g of FeCl_3_·6H_2_O was dissolved in 30 ml of water, and then a mixture solution (ethanol, 40 ml; hexane, 70 ml; oleic acid, 9.5 ml) was added and stirred for 40 min. Next, 0.24 g of NaOH was added to the mixture and stirred for 40 min; the resultant mixture was kept at 70 °C for 4 h. Following the completion of the reaction, the organic layer carrying Fe(oleate)_3_ complex was collected and washed with water and dried at 85 °C overnight. The resultant Fe(oleate)_3_ was dispersed in oleic acid (9.6 ml) and 1-Octadecene (62.5 ml) solution at room temperature and degassed by purging with N_2_ for 1 h. Subsequently, the mixture was heated to 280 °C gradually with a rate of 5 °C min-^1^ under N_2_ flow, then remained at 320 °C for 1 h. The resulting solution was cooled to room temperature and precipitated by adding 500 ml of acetone and centrifuged at 22,000 ×g for 20 min. Eventually, the precipitated Fe_3_O_4_ nanoparticles were dispersed in chloroform. 

**Synthesis of Fe**_3_ **O**_4_ **@SiO**_2_

A volume of 0.5 ml of synthesized Fe_3_O_4 _nanoparticles (40 mg/ml in chloroform) was added to a 5-ml cetyltrimethyl ammonium bromide solution (55 mM) and stirred vigorously for 45 min. Then the solution was warmed up to 65 °C and kept at 37 °C for 1 h to evaporate chloroform. The obtained solution was added to a mixture (45 ml of water and 0.3 ml of NaOH 0.2 M) and heated up to 75 °C. After 5 min, 0.6 ml of TEOSwas added, and stirred for 5 h. Finally, the synthesized Fe_3_O_4_@SiO_2_ nanoparticles were dispersed in 20 ml of ethanol. 

**Synthesis of Fe**_3_ **O**_4_ **@NiSiO**_3_ **nano-magnetic particles**

**Fig. 1:**
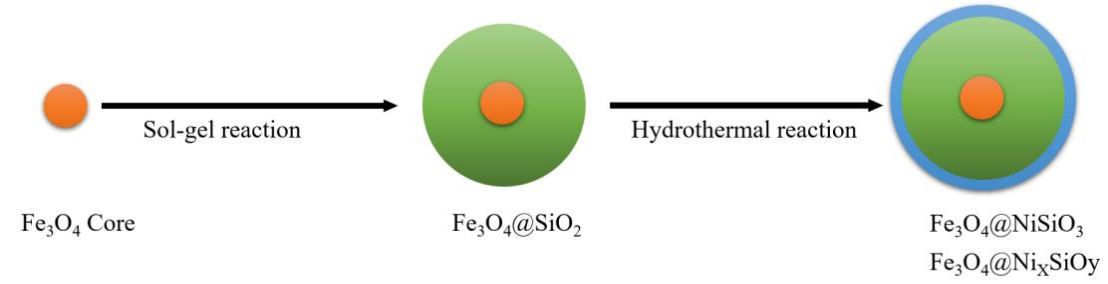
Schematic representation of MNPs synthesis steps

Fe_3_O_4_@NiSiO_3 _was synthesized based on the Wang's method^[^^6^^]^. First, a magnetic core (Fe_3_O_4_) was synthesized as described, and then a SiO_2_ shell was coated on the Fe_3_O_4 _core by Sol-gel procedure (Fig. 1). The synthesized Fe_3_O_4_@SiO_2_ solution was sonicated for 45 min, and mixed with a solution containing NiCl_2_·6H_2_O (133.3 mg), NH_4_Cl (276.5 mg) deionized water (10 ml), ethanol (10 ml), and ammonia solution (1 ml, 28%). The mixture solution was transferred into a Teflon-lined stainless-steel autoclave (50 ml) and sealed to heat at 170 °C for 10 h. Finally, the resulting precipitate was collected by centrifugation (22,000 ×g, 20 min) then washed with deionized water and ethanol and dried at 42 °C overnight. 

**Synthesis of Fe**_3_ **O**_4_ **@Ni**_x_ **SiO**_y_ **nano-magnetic particles**

Fe_3_O_4_@Ni_x_SiO_y_ was synthesized as per Wu's method^[^^13^^]^. Synthesized Fe_3_O_4_ (0.20 g) particles were dispersed in 70 ml of a solution of ethanol-water-ammonia (50:20:1) and stirred vigorously for 1 h. Following that, a mixture solution containing TEOS (2 ml) and ethanol (30 ml) was added gradually by dropping into the above solution. Next, the mixture was heated up to 50 °C for 6 h to achieve Fe_3_O_4_@SiO_2_, and 0.1 g of obtained Fe_3_O_4_@SiO_2_ was added to 10 ml of Ni^+2^ solution containing NiCl_2_6H_2_O (2 mmol) and NH_3_H_2_O (2.5 ml). Subsequently, the mixed solution was transferred to a Teflon-lined stainless steel autoclave and heated at 110 °C for 12 h^[^^17^^]^. After completing the reaction, the Fe_3_O_4_@Ni_x_SiO_y_ MNPs were collected by a neodymium magnet.

**Characterizations of the** **MNPs**

The structural properties of the NMPs analyzed by XRD with a X'Pert-PRO advanced diffractometer using Cu (Kα) radiation (wavelength: 1.5406 Å) were operated at 40 kV and 40 MA at room temperature with 2θ intervals. The morphological characteristics and shape of Fe_3_O_4_@SiO_2 _and Fe_3_O_4_@Ni_x_SiO_y _MNPs were identified by SEM using a Philips XL30 ESEM microscope at an accelerating voltage of 5 kV. The magnetic features of the MNPs were identified through VSM (Meghnatis Kavir Kashan Co., Kashan, Iran) at room temperature.

**Plasmid construction**

All of the cloning steps were performed in Top10 *E. coli *(Invitrogen™, USA) using heat shock method based on the standard protocols^[^^18^^]^. In order to clone and express recombinant SK-His, the SK gene fragment was amplified by specific primers containing the *Nde*I and *Xho*I restriction sites (primers 1 and 2, Table 1) using genomic DNA of *Streptococcus equisimilis* ATCC 9542, as a template. The PCR product was digested by *Nde*I and *Xho*I enzymes and ligated into pET28a (+) plasmid. For EGFP-His cloning, specific primers containing *Nde*I and *Xho*I restrictions sites (primers 3 and 4, Table 1) were used to amplify EGFP gene using pcDNA3-EGFP, as a template. After digestion by the mentioned enzymes, the amplified EGFP was cloned into the pET28a (+) plasmid. Both constructs were confirmed by restriction enzyme analysis.

**Protein expression**

The confirmed constructs containing EGFP and SK were separately transformed to* E. coli* BL21 (DE3; Invitrogen™, USA) competent cells using heat shock method according to standard protocols^[^^18^^]^. Clone selection was performed on Luria-Bertani agar plate containing 50 mg/ml of kanamycin after 18-h incubation at 37 °C. Expression of SK-His and EGFP-His was induced by adding IPTG at the final concentration of 0.8 mM at 16 °C for 20-22 h. Cells were harvested at 15,000 ×g at 4 °C for 20 min and stored at -80 °C. The harvested cells were resuspended in a 30-ml lysis buffer (stated separately for EGFP-His and SK-His, and then disrupted by sonication (Q125 sonicator, Misonix, USA) at Amp 50, with a 15 s pulse, 25 s pause on ice for 15 pulses. The solubilized proteins were separated by centrifugation (15,000 g for 20 min), and the clarified cell lysate was used for further purification steps. Final purified EGFP-His and SK-His concentrations were determined by micro-Bradford assay according to the standard protocols, using bovine serum albumin (0.5-60 µg/ml) as standard^[^^19^^]^. SDS- PAGE densitometry analysis was performed by ImageJ software (version 1.51n) for semi-quantitative protein assays. 

**Purification of EGFP-His and SK-His by** **MNPs**

SK-His and EGFP-His were purified under denaturing and native conditions, respectively. In brief, the frozen cell pellet from SK-His preparation was resuspended in denaturing binding buffer containing 8 M of urea, 100 mM of NaH_2_PO_4_, 100 mM of Tris-Cl, pH 8.0, and sonicated as described before. The solubilized inclusion bodies were mixed with 20 mg of MNPs and incubated at room temperature for 30 min with gentle shaking. The MNP-trapped His-tagged SK was collected by the neodymium external magnetic force. After three washes with wash buffer (8 M of urea, 20 mM of NaH_2_PO_4_, and 500 mM of NaCl, pH 6.0), the fusion proteins were eluted using an elution buffer (6 M of urea, 100 mM of NaH_2_PO_4_, and 100 mM of Tris-HCl, pH 4.5), and then the MNPs were collected by the neodymium external magnetic force. 

**Table 1:** Primers sequences for gene construction

**No** **.**	**Primer name**	**Sequence (5’-3’)**
1	SK-His F *Nde*I	ATACATATGATTGCTGGACCTGAGTG
2	SK-His R *Xho*I	ATATCTCGAGTTTGTCGTTAGGGTTATCAG
3	EGFP-His F *Nde*I	ATACATATGATGGTGAGCAAGGGCGAGG
4	EGFP-His R *Xho*I	ATACTCGAGCTTGTACAGCTCGTCCATGC

Restriction enzymes sites are underlined.

**Fig. 2:**
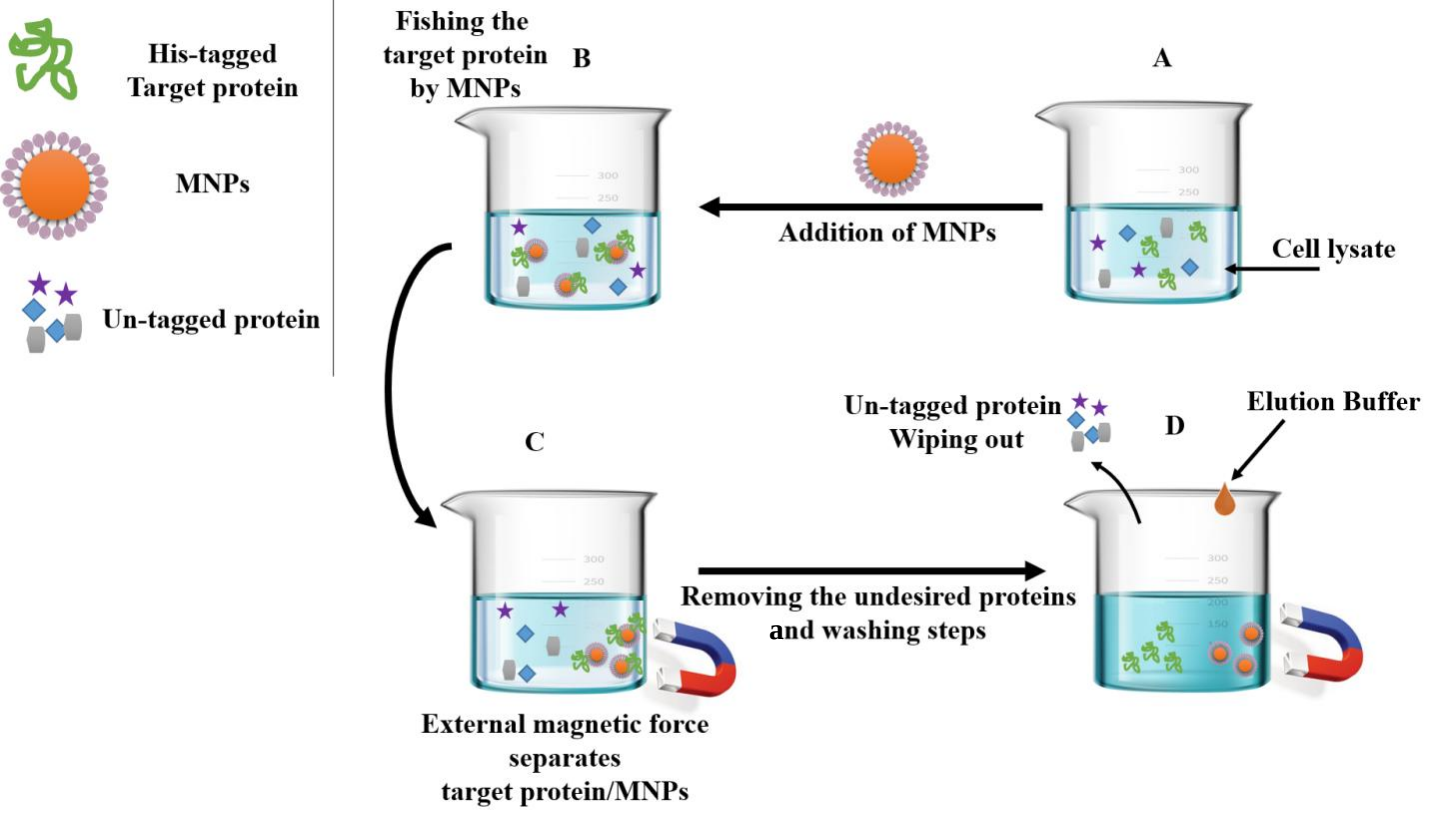
Schematic representation of protein purification by MNPs. (A) MNPs added to cell lysate containing the His-tagged target protein and untagged protein, (B) MNPs traped the His-tagged target protein, (C) His-tagged target protein/MNPs complex collected by the external magnetic force, and (D) un-tagged proteins removed after wash steps and the His-tagged target protein release by the addition of imidazole

In order to purify the EGFP-His, cell lysate was resuspended in a binding buffer (10 mM of imidazole, 50 mM of NaH_2_PO_4_, and 0.5 M of NaCl, pH 8.0), mixed with 20 mg of MNPs and incubated at room temperature for 30 min with gentle shaking. The washing step was performed by 8 ml of wash buffer (40 mM of imidazole, 50 mM of NaH_2_PO_4, _and 0.5 M of NaCl, pH 8). Subsequently, the trapped EGFP-His was collected by an elution buffer (500 µl:500 mM of imidazole, 50 mM of NaH_2_PO_4_, and 0.5 M of NaCl, pH 8) for four times, and finally, the MNPs were collected by the neodymium external magnetic force (Fig. 2). 

**SDS-PAGE and Western blot analyses**

To evaluate the protein expression and identification of purified proteins, SDS-PAGE was carried out according to the standard protocols and Coomassie blue staining (G250)^[^^18^^]^. Protein identification was conducted by Western blot analysis; the recombinant proteins were transferred to a nitrocellulose membrane, which was detected by horseradish peroxidase-conjugated anti-6×-His-tag® monoclonal antibody (BioLegend, USA). Protein bands were finally visualized by brief exposure to 3,3’-diaminobenzidine (Qiagen, USA).

## RESULTS

**Characterizations** **of Fe**_3_ **O**_4_ **@NiSiO**_3_ **and** **Fe**_3_ **O**_4_ **@Ni**_x_ **SiO**_y_

**XRD** **results**

**Fig. 3:**
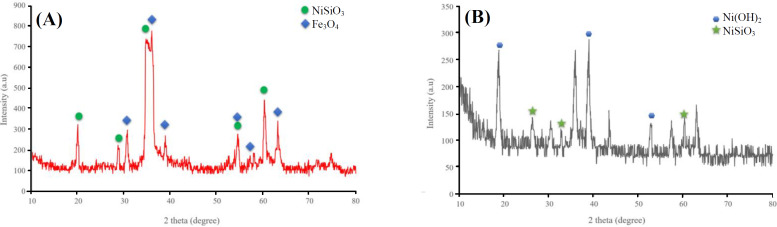
XRD patterns of nanoparticles. (A) the XRD pattern of Fe3O4@NiSiO3 and Fe3O4@NixSiOy with corresponding picks for NiSiO3 and Fe3O4 and (B) the XRD pattern of for Ni (OH)2 and NiSiO3 , respectively

Figure 3 shows XRD crystallographic structures of Fe_3_O_4_@NiSiO_3_ and Fe_3_O_4_@Ni_x_SiO_y _MNPs. As shown in the Figure, both MNPs represent face-centered cubic structures for the Fe_3_O_4 _in their structures (JCPDS 19-0629)^[^^20^^,^^21^^]^. Besides, nickel silicate crystal is present in the Fe_3_O_4_@NiSiO_3 _and Fe_3_O_4_@Ni_x_SiO_y_ structures considering the diffraction peaks in the pattern for the MNPs (JCPDS 43-0664)^[^^22^^]^. Diffraction peak corresponding to nickel hydroxide is determined in Fe_3_O_4_@Ni_x_SiO_y _XRD pattern (JCPDS 73-1520; Fig. 3B)^[^^13^^]^.

**SEM results**

**Fig. 4:**
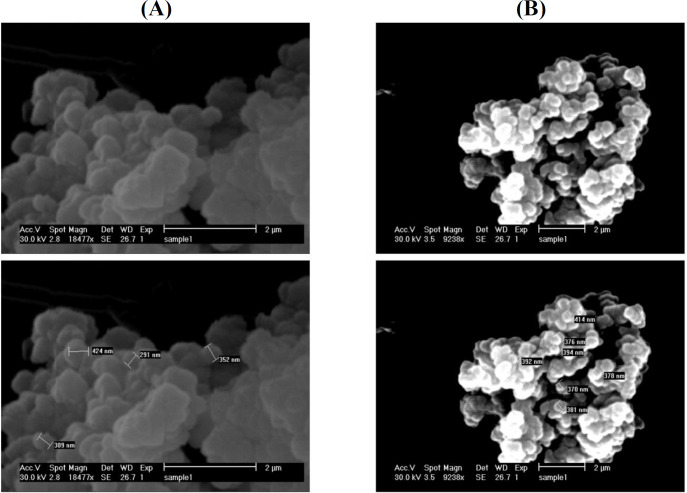
The SEM image of MNPs with the measured scale from (A) Fe_3_O_4_@NiSiO_3_ and (B) Fe_3_O_4_@Ni_x_SiO_y_ MNPs

The SEM images in Figure 4 illustrate the spherical shape for both Fe_3_O_4_@NiSiO_3_ and Fe_3_O_4_@Ni_x_SiO_y _MNPs with the widely different sizes about 330 ± 35 nm (Fig. 4A) for the former and about 370 ± 40 nm (Fig. 4B) for the latter.

**Magnetization properties result by** **VSM**

**Fig. 5:**
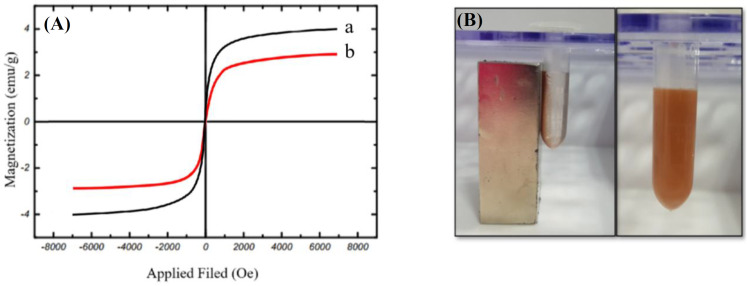
Magnetic properties of the synthesized MNPs (A) VSM result of magnetic separation and redispersion process of the MNPs in PBS. (A) VSM results for (a) Fe_3_O_4_@NiSiO_3_ and (b) Fe_3_O_4_@Ni_x_SiO_y_. (B) Fe_3_O_4_@NiSiO_3_ MNPs in disperse form and in the presence of a magnetic force

As shown in Figure 5, the obtained magnetization curve for both Fe_3_O_4_@NiSiO_3_ and Fe_3_O_4_@Ni_x_SiO_y_ MNPs show superparamagnetic properties, which suggest that magnetic remanence and coercive force are zero. The specific magnetization saturation values were 4.02 emu/g and 2.91 emu/g for Fe_3_O_4_@NiSiO_3_ and Fe_3_O_4_@Ni_x_SiO_y_, respectively, indicating a suitable magnetic property for both MNPs in the presence of an external magnetic force.

**His-tagged protein purification by** **Fe**_3_ **O**_4_ **@NiSiO**_3_ **and** **Fe**_3_ **O**_4_ **@Ni**_x_ **SiO**_y_

**Fig. 6:**
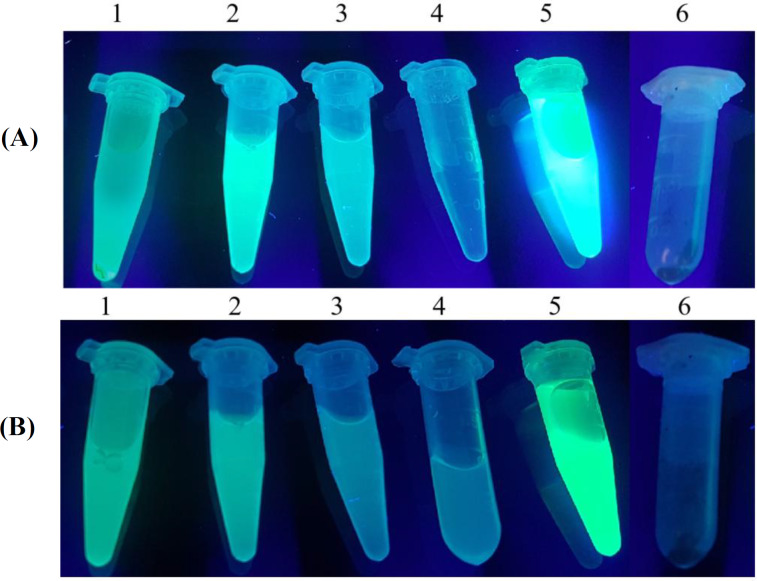
EGFP-His trapping by MNPs visualized by ultraviolet light (UV 538 nm). (A) 1,Un-attached EGFP-His and cell lysate; 2-4, 1st to 3rd wash of the Fe_3_O_4_@NiSiO_3_ MNPs; 5, final elution by imidazole (300 mM); 6, Fe_3_O_4_@NiSiO_3_ MNPs after the purification process. (B) 1, Un-attached EGFP-His and cell lysate; 2-4, 1st to 3rd wash of the Fe_3_O_4_@Ni_x_SiO_y_ MNPs; 5, final elution by imidazole (300 mM); 6, Fe_3_O_4_@Ni_x_SiO_y_ MNPs after the purification process

**Fig. 7:**
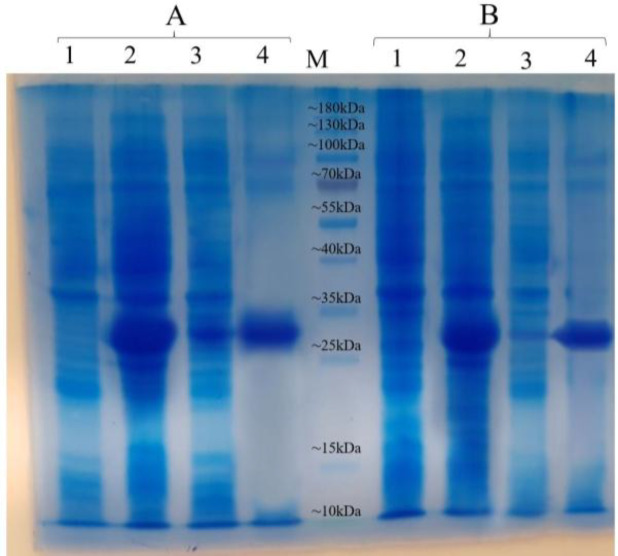
SDS-PAGE result for EGFP-His purification by the MNPs under native conditions. EGFP-His purification via (A) Fe_3_O_4_@NiSiO_3_ and (B) Fe_3_O_4_@NiSiO_3_ MNPs: lanes 1, un-induced cell lysate; lanes 2, induced cell lysate after 22 h; lanes 3, cell lysate after purification by Fe_3_O_4_@NiSiO_3_ and Fe_3_O_4_@Ni_x_SiO_y_ MNPs; lane 4, purified EGFP-His by Fe_3_O_4_@NiSiO_3_ and Fe_3_O_4_@Ni_x_SiO_y_ MNPs in final elution via imidazole; M, protein marker

**Fig. 8:**
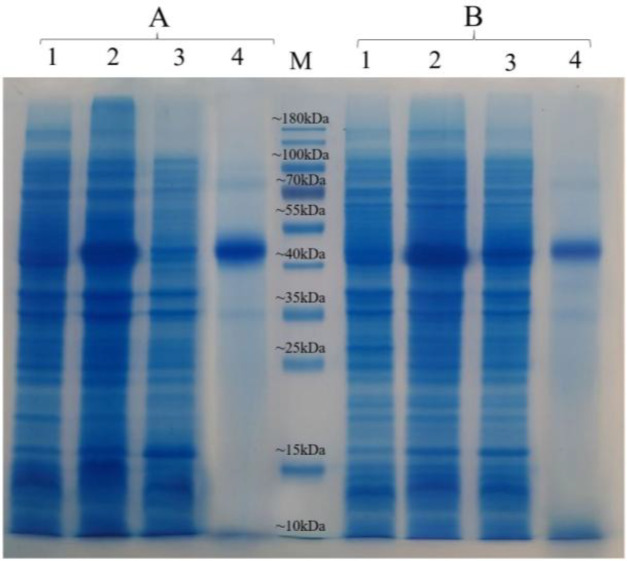
SDS-PAGE result for SK-His purification by the MNPs under the denature conditions. SK-His purification via (A) Fe_3_O_4_@NiSiO_3_ and (B) Fe_3_O_4_@Ni_x_SiO_y_ MNPs: lanes 1, un-induced cell lysate; lanes 2, induced cell lysate after 22 h; lanes 3, cell lysate after purification by Fe_3_O_4_@NiSiO_3_ and Fe_3_O_4_@Ni_x_SiO_y_ MNPs; lanes 4, purified SK-His by Fe_3_O_4_@NiSiO_3_ and Fe_3_O_4_@Ni_x_SiO_y_ MNPs in elution buffer; M, protein marker

**Fig. 9:**
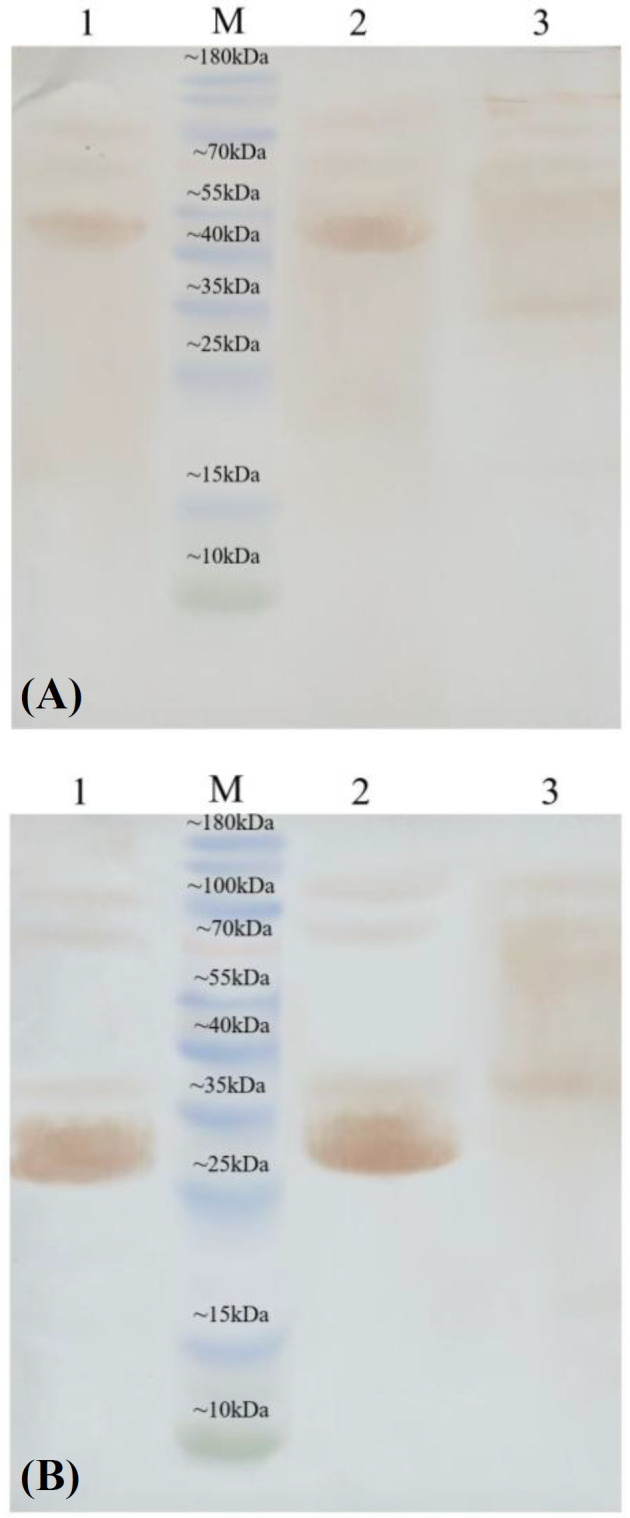
Western Blot result for SK-His and EGFP-His purified by the synthesized MNPs. Western blot result for purified (A) SK-His and (**B) **EGFP-His under the denature conditions: lanes 1, purified SK-His and EGFP_His by Fe_3_O_4_@NiSiO_3_ MNPs; lanes 2, purified SK-His by Fe_3_O_4_@Ni_x_SiO_y_ MNPs; lane 3, un-induce cell lysate. M, protein marker

As illustrated in Figure 6, both Fe_3_O_4_@NiSiO_3_ and Fe_3_O_4_@Ni_x_SiO_y _MNPs successfully purified EGFP-His, directly from the cell lysate. Binding capacities for both MNPs were measured after the addition of the MNPs to an excessive amount of cell lysate containing EGFP-His. The results indicated that the Fe_3_O_4_@NiSiO_3_ MNPs were able to capture EGFP-His at 16565 ± 8 µg per 80 mg of MNPs (207 µg/mg). This amount was 15605 ± 6 µg per 80 mg of Fe_3_O_4_@Ni_x_SiO_y_ MNPs (195 µg/mg). All measurements were in triplicates (Table 2). Samples from different steps of the purification process (Fig. 2) of EGFP-His were loaded on SDS-PAGE for further analysis and confirmed by Western blot. As shown in Figure 7 and Figure 9B , a sharp protein band is apparent between 25 kDa and 35 kDa positions of the protein marker, which corresponds to EGFP-His (30 kDa). The purity percentages of both MNPs was calculated by ImageJ software (version 1.51n), and the result represented more purity of Fe_3_O_4_@NiSiO_3 _than Fe_3_O_4_@Ni_x_SiO_y_ (Table 2). Purified SK-His by the two MNPs was loaded on SDS-PAGE for evaluating the quality of purification process. As shown in Figures 8 and 9A, a sharp and specific protein band is visible around 47 kDa. The SK-His purity percentages of both MNPs calculated by ImageJ software revealed almost the same purity percentage for both synthesized MNPs (Table 2). As shown in Figure 9, Western blotting analyses of the purified EGFP-His and SK-His confirmed the validity of the purified protein by both Fe_3_O_4_@NiSiO_3 _and Fe_3_O_4_@Ni_x_SiO_y_ MNPs.

**Table 2:** Protein purification and quantification via MNPs

**Protein**	**Purification conditions**	**MNP**	**Yield** ^a^ **(** **µ** **g/mg)**	**Standard deviation (** **µ** **g/mg)**	**Purity ** **(%)** ^b^
SK-His	Denature	Fe_3_O_4_@NiSiO_3_	192	±4.4	~81
		Fe_3_O_4_@Ni_x_SiO_y_	188	±3.4	~80
					
EGFP-His	Native	Fe_3_O_4_@NiSiO_3_	207	±3.9	~73
		Fe_3_O_4_@Ni_x_SiO_y_	195	±4.1	~71

All values and errors were represented as mean and standard deviations, respectively, from three independent purification experiments. ^a^Purification yields were determined using 80 mg of MNPs; ^b^Purity percentage was estimated using densitometry analysis on SDS-PAGE.

## DISCUSSION

In the current study, we have synthesized two MNPs with the magnetic core of Fe_3_O_4_, SiO_2 _shell, and immobilized Ni^2+^ on the surface to examine the capability of the MNPs for His-tagged protein purification from inclusion bodies. The inclusion bodies form of SK-His was purified successfully beside the soluble EGFP-His as the model proteins. Purification of EGFP-His and SK-His under native and denature conditions demonstrated an average purity of 72% and 80%, respectively Evaluation by XRD (Fig. 3), SEM (Fig. 4), and VSM (Fig. 5) of both Fe_3_O_4_@NiSiO_3 _and Fe_3_O_4_@Ni_x_SiO_y_ MNPs confirmed their structure, morphology, size, and magnetization properties the same as the previous reports^[^^6^^,^^13^^]^. The measured Fe_3_O_4_@NiSiO_3 _MNPs binding capacity for EGFP-His (30 kDa) was 207 µg/mg, which was comparable with Wang *et al.*^[^^6^^]^result (220 µg/mg). However, Fe_3_O_4_@Ni_x_SiO_y _represented 195 µg/mg binding capacity, which was similar to the result obtained by Wu and co-workers^[^^13^^]^ (193 µg/mg). More than 70% purity for both MNPs was obtained (Table 2), which is a suitable purity rate under the native conditions. However, buffer optimization and the increase of the total amount of immobilized Ni^2+^ on the MNPs surface could lead to more purity percentages.

Inclusion body expression is a well-known strategy for SK production^[^^15^^]^; therefore, it was used as a model protein for inclusion body purification under the denaturing conditions. Fe_3_O_4_@NiSiO_3 _and Fe_3_O_4_@Ni_x_SiO_y_ MNPs were represented purification capability under the denaturing conditions with the yield of 192 µg/mg and 188 µg/mg, respectively (Table 2). Despite the fewer yields as compared to EGFP-His, the average purity percentage obtained by both MNPs under the denaturing conditions was higher than that of EGFP-His (80% vs. 72%). Harsh denaturing conditions unfolds the proteins structure; consequently, unspecific attachment to the MNPs decreases, and fusion His tag can easily binds to immobilized Ni on the surface of MNPs. MNPs Fe_3_O_4_/PMG/IDA-Ni^2+^ (103 μg/mg)^[^^23^^]^, Fe_3_O_4_Au-ANTA-Co^2+^ (74 µg/mg)^[^^12^^]^, and chitosan/ Fe_3_O_4_ (62.8 µg/mg)^[^^24^^]^ with different kinds of conjugated groups and different binding capacities have been reported. However, the binding capacities of these MNPs may be affected under harsh denaturing conditions due to the complexes in their structures. 

In conclusion, MNPs with a magnetic core of Fe_3_O_4_, SiO_2 _shell, and immobilized Ni^2+^ on the surface (Fig. 1) can purify His-tagged protein from inclusion bodies approximately up to 80%. The binding capacities for both synthesized Fe_3_O_4_@NiSiO_3 _and Fe_3_O_4_@Ni_x_SiO_y_ MNPs were suitable and comparable with their performance under the native conditions. Low-cost production along with high binding capacity and purity percentage makes Fe_3_O_4_@NiSiO_3 _and Fe_3_O_4_@Ni_x_SiO_y_ MNPs attractive choices for His-tagged protein purification from inclusion bodies. 
